# Disparity of perception of quality of life between head and neck cancer patients and caregivers

**DOI:** 10.1186/s12885-021-08865-7

**Published:** 2021-10-20

**Authors:** Zachary M. Kassir, Jinhong Li, Christine Harrison, Jonas T. Johnson, Marci L. Nilsen

**Affiliations:** 1 Department of Otolaryngology, University of Pittsburgh School of Medicine, Eye & Ear Institute, 203 Lothrop Street, Pittsburgh, PA 15213 USA; 2 Department of Biostatistics, University of Pittsburgh Graduate School of Public Health, 130 De Soto Street, Pittsburgh, PA 15261 USA; 3 Department of Acute and Tertiary Care, University of Pittsburgh School of Nursing, 318A Victoria Building, 3500 Victoria Street, Pittsburgh, PA 15261 USA

**Keywords:** Caregiver, Caregiver distress, Caregiver burden, Head and neck, Quality of life

## Abstract

**Background:**

Caregivers are invaluable sources of support for individuals recovering from head and neck cancer (HNC). Accordingly, minimizing caregiver distress is essential to promote the well-being of both caregivers and their patients. This study assessed if psychosocial distress (i.e., anxiety and depression) among HNC caregivers is associated with a difference in how caregivers and their patients perceive patients’ quality of life (QOL) after treatment completion.

**Methods:**

Caregivers’ and patients’ perceptions of patient QOL were assessed using the University of Washington QOL Questionaire (UWQOL), a validated HNC-specific health-related QOL questionnaire. The survey is interpreted in terms of its two composite scores: a physical QOL score and a social-emotional QOL score with higher scores indicating better QOL. Caregiver anxiety was assessed using the Generalized Anxiety Questionaire-7 (GAD-7) and caregiver depression was assessed using the Patient Health Questionaire 8 (PHQ-8). Patients completed the UWQOL as part of clinic intake while caregivers were asked to complete the UWQOL for their patients in addition to the PHQ-8 and GAD-7 in private. Linear regression was used to analyze the association between differences in caregivers’ and patients’ QOL scores (both social-emotional and physical QOL subscale scores) and GAD-7 and PHQ-8 scores.

**Results:**

Of 47 caregivers recruited, 42.6% (*n* = 20) viewed patients’ social-emotional QOL more negatively than patients themselves, while 31.9% viewed patients’ physical QOL more negatively. After controlling for covariates, differences in perception of social-emotional QOL (*p* = .01) and differences in perception of physical QOL (*p* = .007) were significantly associated with caregiver depression, but not anxiety. Caregivers who disagreed with patients regarding patients’ social-emotional QOL scored 6.80 points higher on the PHQ8 than agreeing caregivers. Caregivers who disagreed regarding patients’ physical QOL scored 6.09 points higher.

**Conclusion:**

Caregivers commonly view patients’ QOL more negatively than patients themselves. These caregivers tend to have greater psychosocial distress than caregivers who agree with their patients. Interventions designed to identify and aid at-risk caregivers are critically needed. We propose screening for differences in perception of patient QOL as a way of identifying distressed caregivers as well as provider-facilitated communication between patients and caregivers as possible interventions that should be examined in future research.

**Supplementary Information:**

The online version contains supplementary material available at 10.1186/s12885-021-08865-7.

## Background

According to the American Society of Clinical Oncology, more than 60,000 people will develop head and neck cancer (HNC) in 2021 [1]. HNC is often treated with multiple modalities (i.e., surgery, chemotherapy, radiation), and severe side effects often contribute to long-term morbidity in the post-treatment “survivorship” phase [1, 2]. For example, surgical resection followed by reconstruction can result in disfigurement as well as prolonged pain and limited mobility of the shoulder and neck [2, 3]. Additionally, radiation can destroy major salivary glands and cause oropharyngeal fibrosis, resulting in long-term dysphagia, dysphonia, and aspiration-induced pneumonia [4]. Deficits in sensation and taste are also common after treatment and can be permanent [2, 4].

For illnesses with high morbidity, the presence of an informal caregiver (e.g., a family member or friend) who provides care and assists in the recovery process improves outcomes for patients [5–7]. Among HNC caregivers, commonly reported responsibilities include providing emotional support (in the form of spiritual aid or assuaging patient’s concerns about their appearance), instrumental support (assisting with finances, transportation to appointments, cooking, and housekeeping), and tasks unique to HNC (special food preparation, feeding tube assistance, and acting as patient’s speech assistant) [8]. Meeting these basic necessities is essential for patients in the post-treatment period, making caregivers crucial to the long-term well-being of HNC patients.

In combination with the emotional challenge of coping with a loved one’s illness, the time and effort required to provide care can take an immense toll on caregiver well-being. According to the findings of Hanly et al. as well as Badr et al., many caregivers carry out their duties at the expense of self-care (e.g., missed doctor's appointments, lack of exercise) [9, 10]. Furthermore, in a qualitative cross-sectional study of 31 long-term HNC caregivers, Balfe et al. report that personal sacrifices are accentuated by pervasive feelings of loss regarding social lives, freedom, the appearance and capabilities of their loved ones, and joy in general [11]. Predictably, the existing literature has demonstrated that HNC caregivers endure higher levels of psychological distress than the general population [7] and patients themselves [1, 12, 13]. Moreover, the negative impact on caregivers extends to patients as well. Existing research on caregiving for Alzheimer’s patients has demonstrated that increased levels of “caregiver burden” (i.e., how encumbered caregivers feel by their responsibilities) and psychological distress are associated with patient use of healthcare services and the likelihood of placement in nursing homes [14–16].

There is a crucial need to identify contributing factors to caregiver distress and ways to identify at-risk caregivers. However, at present, the topic remains understudied in the context of HNC. In fields where caregiver distress is more extensively studied, including Alzheimer’s disease, stroke recovery, pain management, and other forms of cancer, studies have examined how differences in perception between patients and caregivers influence caregiver distress. Specifically, it has been demonstrated that caregivers who overestimate the severity of an illness’s impact on patient quality of life (QOL), relative to patient-reported severity, have worse psychological and physical health [17–24]. Accordingly, we are interested to see if this translates to HNC as well. Specifically, we seek to examine the correspondence in how patients and caregivers perceive patients’ HNC-related QOL after treatment completion and if a difference is associated with increased levels of distress in caregivers. An association, if identified, could serve as a potential target of future interventions to mitigate caregiver distress.

## Methods

We conducted a cross-sectional analysis of 47 patient-caregiver dyads who were recruited at the UPMC HNC Survivorship Clinic between August 2019 and April 2020. Individuals accompanying patients who had completed treatment for squamous cell carcinoma of the oral cavity, oropharynx, larynx, hypopharynx, or an unknown primary were screened for eligibility. Both individuals had to be over 18 years of age and identify English as their primary language. Patients had to identify individuals as caregivers who “significantly assisted them during their treatment and recovery” for at least an hour a week. Additionally, patients were asked if the accompanying individual helped them fill out pre-intake surveys. If help was given, dyads were excluded. Caregivers were additionally asked if they had been providing care to the patient since the patient completed treatment or if they started providing care at a later point in the patient’s post-treatment survivorship course. Caregivers completed questionnaires in unoccupied examination rooms to ensure that patients did not directly influence their answers. Researchers remained outside of rooms but were available to answer questions or provide technical support. All methods were performed in accordance with the Declaration of Helsinki. This study was reviewed by the Human Research Protection Office of the University of Pittsburgh who granted approval of all human procedures performed (Study 19,060,320). All participants gave informed consent for this study.

### Caregivers characteristics

Caregivers were asked to complete three questionnaires: Patient Health Questionaire-8 (PHQ-8), Generalized Anxiety Disorder Questionaire-7 (GAD-7), and the University of Washington Quality of Life Questionnaire (UWQOL). Caregivers also answered questions regarding demographics (e.g., age, gender, marital status, education, and employment) and aspects of the caregiving experience (e.g., hours a week and duration of caregiving).

The PHQ-8 [25] and the GAD-7 [26] were completed to assess caregivers’ symptoms of depression and anxiety, respectively. The PHQ-8 is an 8-item questionnaire with scores ranging from 0 to 24, while the GAD-7 has 7 items with scores ranging from 0 to 21. Higher scores indicate more significant symptoms of depression/anxiety. Both questionnaires are well-validated instruments that are used widely in oncologic practice [27, 28].

The UWQOL is a validated HNC-specific health-related QOL questionnaire. It contains twelve domains and is interpreted in terms of its two composite scores: a physical QOL score (i.e., an average of chewing, swallowing, speech, taste, saliva, and appearance domains) and a social-emotional QOL score (i.e., an average of anxiety, mood, pain, activity, recreation, and shoulder domains). Scores range from 0 to 100, with higher scores indicating better QOL [29]. Caregivers were asked to answer this survey for their patients but were asked to select responses that they believe their patients *should* give regardless of the responses that they believe their patients *would* give.

### Patient clinical characteristics

Clinical characteristics of patients, including tumor site, tumor stage, and treatment modality, were abstracted from the medical record. Patients routinely complete the UWQOL as part of the intake for the clinic. These UWQOL scores were obtained from the medical record and were subtracted from caregiver scores. Prior research has established that differences of 7 points or greater in UWQOL scores should be interpreted as clinically significant [30–32]. As such, any caregivers who reported scores at least 7 points less than their patients were identified as perceiving their patients’ QOL to be worse than patients themselves.

### Statistical analysis

Statistical analysis was performed using RStudio (1.1.456; Rstudio, Inc., Boston, Massachusetts). In the descriptive analysis, frequencies and percentages were calculated for categorical variables and means and standard deviations for continuous variables. In univariate analysis, Wilcoxon signed-rank and Kruskal-Wallis rank sum tests were performed to determine the impact of patient clinical characteristics and caregiver characteristics on GAD-7 and PHQ-8 scores for categorical variables with 2 categories or more than 2 categories, respectively. Characteristics that met the screening criteria of *p* ≤ .10 were then considered jointly via multivariate analysis. More specifically, multivariate models were constructed: one that assessed relationships between variables of interest and GAD-7 scores and another that assessed relationships between variables of interest and PHQ-8 scores. The primary variables of interest (i.e., significant differences in perception of physical QOL and significant differences in perception of social-emotional QOL) were included in both models. Interaction plots were used to evaluate potential interactions between variables of interest (i.e., differences in social-emotional QOL and differences in physical QOL). In this way, multivariate linear regression analysis was performed to assess the relationship between significant differences in perception of QOL and GAD-7 and PHQ-8 scores while controlling for covariates that met our screening criteria.

## Results

### Patient and caregiver characteristics

In total, 47 patient-caregiver dyads were included. Table 1 presents caregiver characteristics, while Table 2 presents the clinical characteristics of patients. Mean [Standard deviation (SD)] ages of patients and caregivers were 66.8 (8.6) and 62.6 (10.3), respectively. Caregivers were predominantly female (*n* = 33, 70.2%) and spouses or partners of the patient (*n* = 39, 83.0%). All caregivers had been providing care to their patients since the time of treatment completion. The majority of caregivers had been providing care for more than 12 months (*n* = 33, 70.2%), and most caregivers were providing care for at least 9 h per week (*n* = 32, 68.1%).

**Table 1:** Caregiver characteristics/demographics

	N(%)	Mean GAD-7 Score (SD)	*P* value* (GAD-7 Score)	Mean PHQ-8 Score (SD)	*P* value* (PHQ-8 Score)
**Gender**					
Male	14 (29.8%)	3.27 (5.40) (5.40)	0.05	3.82 (5.13)	0.20
Female	33 (70.2%)	5.25 (4.39)		5.64 (5.21)	
**Marital Status**					
Married/Living with partner	39 (83.0%)	4.72 (4.47)	0.97	4.85 (4.92)	0.28
Not Married	8 (17.0%)	5.13 (5.84)		7.00 (6.41)	
**Education**					
Bachelor’s or beyond	12 (25.5%)	4.92 (4.98)	0.95	4.00 (4.40)	0.30
Less than bachelors	35 (74.5%)	4.74 (4.62)		5.63 (5.44)	
**Employment**					
Working FT/PT	14 (29.8%)	5.71 (5.55)	0.15	5.07 (5.18)	0.13
Not employed or not seeking employment (homemaker or disabled)	12 (25.5%)	6.42 (5.23)		7.25 (5.69)	
Retired	21 (44.7%)	3.24 (3.19)		4.14 (4.80)	
**Difficulty paying for basic needs**					
No	36 (76.6%)	4.53 (4.67)	0.44	4.72 (5.26)	0.10
Yes	11 (23.4%)	5.64 (4.76)		6.82 (4.83)	
**Hours spent caregiving per week**					
8 or less hours per week	15 (31.9%)	3.20 (3.82)	0.16	4.27 (4.20)	0.77
9–39 h per week	13 (27.7%)	6.38 (5.49)		6.38 (6.68)	
40 or more hours per week	19 (40.4%)	4.95 (4.49)		5.16 (4.87)	
**How long have you been giving care**					
< 12 months	14 (29.8%)	5.29 (5.28)	0.91	4.86 (4.91)	0.85
13 monts-5 years	14 (29.8%)	3.93 (3.29)		5.00 (3.46)	
More than 5 years	19 (40.4%)	5.05 (5.17)		5.63 (6.52)	

*Abbreviations*: *GAD-7* Generalized Anxiety Disorder Questionaire-7, *PHQ-8* Patient Health Questionaire-8

*P-values were calculated with Wilcoxon signed-rank test for two-category data and Kruskal-

Wallis rank sum test for data with more than two categories

**Table 2:** Patient Clinical Characteristics

	N(%)	Mean GAD-7 Score (SD)	*P* value* (GAD-7 Score)	Mean PHQ-8 Score (SD)	*P* value* (PHQ-8 Score)
**Tumor Location**					
Oral cavity/maxillary	11 (23.4%)	3.09 (3.53)	0.26	3.73 (4.03)	0.60
Oropharynx	13 (27.7%)	7.08 (5.68)		6.77 (6.70)	
Larynx/Hypopharynx	14 (29.8%)	4.57 (4.54)		5.71 (5.20)	
Other	9 (19.1%)	3.89 (3.76)		4.00 (3.81)	
**Tumor Stage**					
Stage I-II	18 (38.3%)	4.28 (4.07)	0.40	5.33 (5.59)	0.86
Stage III-IV	24 (51.1%)	5.79 (5.26)		5.50 (5.36)	
Unknown Primary	5 (10.6%)	1.80 (1.48)		3.40 (2.61)	
**Treatment**					
Surgery Alone	3 (6.4%)	2.00 (2.00)	0.11	2.67 (0.58)	0.25
RT/CRT	13 (27.7%)	7.54 (6.08)		7.00 (6.14)	
Surgery and RT/CRT	31 (65.9%)	3.90 (3.66)		4.71 (4.89)	

*Abbreviations*: *GAD-7* Generalized Anxiety Disorder Questionaire-7, *PHQ-8* Patient Health Questionaire-8

*P-values were calculated with Wilcoxon signed-rank test for two-category data and Kruskal-

Wallis rank sum test for data with more than two categories

The larynx/hypopharynx was the most common tumor site (*n* = 14, 29.8%), though the distribution of patients was largely equitable across all sites. Half of the patients were diagnosed with advanced-stage (III-IV) disease (*n* = 24, 51.1%), and most were treated with both surgery and adjuvant treatment (*n* = 31, 65.9%).

Mean (SD) caregiver GAD-7 and PHQ-8 scores were 4.79 (4.66) and 5.21 (5.19), respectively. Caregiver gender was the only variable that met the criteria (*p* ≤ 0.1) for inclusion in our multivariate model for GAD-7 scores, with female caregivers and male caregivers reporting mean (SD) GAD-7 scores of 5.25 (4.39) and 3.27 (5.40), respectively (*p* = .05). Difficulty paying for basic needs was the only variable that met the criteria for inclusion in our multivariate model for PHQ-8 scores with caregivers who admitted to difficulty and caregivers denying difficulty reporting mean (SD) PHQ-8 scores of 6.82 (4.83) and 4.72 (5.26), respectively (*p* = 0.1).

### Caregivers’ perception of patient quality of life

For patients, the mean physical QOL score was 64.68 (SD = 19.22), and the mean social-emotional QOL score was 73.55 (SD = 18.84). Mean physical and social-emotional QOL scores reported by caregivers were 62.25 (SD = 16.78) and 67.82 (SD = 19.89), respectively. When caregivers and patients agreed on social-emotional QOL, the mean social-emotional QOL scores reported by caregivers and patients were 72.87 (SD = 20.40) and 69.62 (SD = 16.41), respectively. When caregivers and patients disagreed regarding social-emotional QOL, the mean social-emotional QOL scores reported by caregivers and patients were 61.0 (SD = 17.39) and 78.83 (SD = 18.76), respectively. Caregivers and patients who agreed regarding physical QOL reported mean physical QOL scores of 64.30 (SD = 18.25) and 60.10 (SD = 15.91), respectively, while caregivers and patients who disagreed regarding physical QOL reported mean physical QOL scores of 57.89 (SD = 12.54) and 74.4 (SD = 14.92), respectively. Of the 47 caregivers enrolled in this study, 42.6% viewed patient social-emotional QOL more negatively, and 31.9% viewed physical QOL more negatively than patients themselves (Table 3). Fisher’s exact test showed that differences in perception of physical QOL and differences in perception of social-emotional QOL were significantly associated with each other (*p* < .05). An interaction plot (Supplemental Fig. 1) reinforces this conclusion. As such, an interaction term was incorporated into both of our multivariate models.

**Table 3:** Association between GAD-7/PHQ-8 scores and significant differences in perception

	N(%)	Mean GAD-7 Score (SD)	*P* value* (GAD-7 score)	Mean PHQ-8 Score (SD)	*P* value* (PHQ-8 Score)
**Significant difference in perception of social-emotional quality of life (perceived worse by caregiver)**					
Yes	20 (42.6%)	5.90 (4.95)	0.11	7.10 (6.33)	0.07
No	27 (57.4%)	3.96 (4.35)		3.81 (3.69)	
**Significant difference in perception of physical quality of life (perceived worse by caregiver)**					
Yes	15 (31.9%)	6.33 (5.70)	0.21	7.33 (6.28)	0.07
No	32 (68.1%)	4.06 (3.98)		4.22 (4.36)	

*Abbreviation*: *GAD-7* Generalized Anxiety Disorder Questionaire-7, *PHQ-8* Patient Health Questionaire-8

*P-values were calculated with Wilcoxon signed-rank test

Table 4 shows that, when controlling for caregiver gender, differences in perception of social-emotional QOL *and* differences in perception of physical QOL were not significantly associated with caregiver anxiety symptoms (*p* > .05). However, it is worth noting that both variables were just beyond the boundary of significance (*p* = .08 and *p* = .07, respectively). *(Insert* Table 4*)* Table 5 shows that, when controlling for difficulty paying for basic needs, differences in perception of social-emotional QOL *and* differences in perception of physical QOL were significantly associated with PHQ-8 scores (*p* = .01 and *p* = .007, respectively). Caregivers who reported patients to have worse social-emotional QOL than patients themselves had PHQ-8 scores 6.80 points higher than caregivers who agreed with patients. (95% confidence interval [CI], 1.66, 11.49; *p* = .01). Caregivers who reported patients to have worse physical QOL than patients themselves had PHQ-8 scores 6.09 points higher than caregivers who agreed with patients (95% confidence interval [CI], 1.78, 10.40; *p* < .01).

**Table 4:** Multivariate Linear Regression of Variables Associated with GAD-7 Score

Variables	Coefficient (95% CI)	*P* value
**Significant difference in perception of social-emotional quality of life (perceived worse by caregiver)**		0.08
**No**	(Base)	
**Yes**	4.40 (−0.58, 9.38)	
**Significant difference in perception of physical quality of life (perceived worse by caregiver)**		0.07
**No**	(Base)	
**Yes**	3.80 (−0.27, 7.87)	
**Interaction**	4.63 (−1.45, 10.70)	0.13
**Caregiver Gender**		0.24
**Female**	(Base)	
**Male**	−1.86 (−5.00, 1.28)	

*Abbreviation*: *GAD-7* Generalized Anxiety Disorder Questionaire-7, *PHQ-8* Patient Health Questionaire-8

**Table 5:** Multivariate Linear Regression of Variables Associated with PHQ-8 Score

Variables	Coefficient (95% CI)	*P* value
**Significant difference in perception of social-emotional quality of life (perceived worse by caregiver)**		0.01
**No**	(Base)	
**Yes**	6.80 (1.66, 11.94)	
**Significant difference in perception of physical quality of life (perceived worse by caregiver)**		0.007
**No**	(Base)	
**Yes**	6.09 (1.78, 10.40)	
**Interaction**	6.77 (0.52, 13.02)	0.03
**Difficulty paying for basic needs**		0.08
**No**	(Base)	
**Yes**	2.97 (−0.41, 6.35)	

*Abbreviation*: *GAD-7* Generalized Anxiety Disorder Questionaire-7, *PHQ-8* Patient Health Questionaire-8

## Discussion

This is the first study to demonstrate that HNC caregivers who perceive patients’ QOL more negatively than patients themselves after treatment completion have more significant psychological distress. It was found that roughly one-third of caregivers viewed the social-emotional QOL, and almost half viewed the physical QOL of their patients more negatively than patients themselves.

Disagreement regarding physical QOL and disagreement regarding social-emotional QOL were not significantly associated with caregiver anxiety in our multivariate analysis (Table 4). However, disagreement regarding physical QOL and disagreement regarding social-emotional QOL were both significantly associated with caregiver depression (Table 5). On average, caregivers who agreed with patients regarding physical QOL or social-emotional QOL reported PHQ-8 scores that indicated no symptoms of depression. On the other hand, caregivers who disagreed with patients regarding physical or social-emotional QOL reported PHQ-8 scores more than 6 points higher than caregivers who agreed after controlling for covariates. The fact that an association was found with depression but not anxiety is consistent with prior research, including the findings of Lee et al. who found that 6 months after diagnosis of HNC, 12.9% of caregivers had depressive disorders while 0% had anxiety disorders [33].

It is worth noting that among patient-caregiver dyads that disagreed regarding social-emotional QOL (i.e., when caregivers reported scores at least 7 points lower than patients), the actual mean difference in social-emotional QOL scores reported by patients and caregivers was 17.83. When dyads disagreed regarding physical QOL the mean difference in physical QOL scores was 16.51. Future research involving longitudinal studies with larger cohorts should explore if there is a range of cutoffs that define significant differences in perception of QOL and if using different cutoffs may strengthen the association between differences in perception of QOL and caregiver distress.

Our findings are in line with those of Deschler et al., who reported that among a cohort of 14 patient-caregiver dyads, 45% of caregivers rated patient QOL lower using the SF-36 survey 6 months post-operatively [34]. As for the association we observed between differences in perception and caregiver distress, our findings are consistent with the literature in other fields. In a 2016 study of couples living with non-small cell lung cancer, Lyons et al. reported that, on average, spouses rated patients’ fatigue more severely than patients themselves. Moreover, spouse mental health was worse when couples had disparate appraisals of patients’ fatigue [23]. Similarly, in a 2015 study of stroke survivors and their spouses, McCarthy et al. reported that 43% of spouses rated survivor physical-functioning (as measured by the Stroke Impact Scale) significantly more negatively than survivors themselves. Moreover, spouses who perceived their patients’ physical function more negatively than patients themselves were significantly more likely to experience greater depressive symptoms as measured by the PHQ8 [20], which was also identified in our study. Indeed, this association is heavily supported because it does not appear to depend on the instrument used to measure physical function (our study used the UWQOL while McCarthy et al. used the Stroke Impact Scale). Furthermore, Miaskowski et al. reported on patient-caregiver dyads going through cancer treatment who were asked to rate the severity of patients’ pain. According to the authors, caregivers in “non-congruent dyads” had significantly higher depression scores and caregiver strain scores than those in “congruent dyads.” [21] This finding is in line with our study, especially given that “pain” is one of the elements included in the social-emotional QOL score of the UWQOL questionnaire.

We considered multiple explanations for the association between differences in perception of QOL and caregiver distress. For one, we wonder if distressed caregivers are inherently more likely to have more negative views of patient QOL than patients themselves. Simply put, existing literature suggests that HNC caregivers endure higher levels of psychological distress than the general population [7] and patients themselves [1, 12, 13]. This seems intuitive given the burden of the caregiving role. Caregiving responsibilities often come at the expense of self-care [9, 10], and the challenge is made even more difficult by the sense of loss regarding personal freedom, the appearance and health of a loved one, and overall happiness [11]. It is conceivable that these factors elicit caregiver depression which, in turn, negatively skews caregivers’ perceptions regarding patient QOL. As such, relatively negative views of patient QOL may be an accurate indicator of overall caregiver distress.

The idea that differences in perception of QOL indicate generalized distress is supported by existing literature, demonstrating that caregivers have more negative views than patients in other areas. For instance, as reported by Hodges et al., fear of recurrence is typically greater among caregivers than patients during the post-treatment period [35]. Moreover, greater fears of recurrence are predictive of greater future psychological distress. Additionally, Richardson et al. report that caregivers express greater concern about the illness at the time of HNC diagnosis and believe that it will last longer than patients [36, 37]. Simply put, we wonder if these relatively negative perceptions collectively indicate a generally negative outlook that suggests depressive symptoms, which are highly common among HNC caregivers. Future studies could elaborate on this possibility by determining if caregivers with negatively skewed perceptions of one aspect of the illness experience are more likely to have similarly negative views on others and if depressed caregivers are more likely to have negatively skewed views on all topics.

Based on this theory, we propose screening for discrepancies in perception as a way of identifying distressed caregivers. Specifically, comparing caregivers’ perceptions of patients’ QOL against patients’ perceptions is prudent given the existence of a validated HNC QOL instrument (UWQOL) that can quantify and objectively indicate significant disparities in perception. In practice, every time a patient completes the UWQOL to assess for changes in their QOL caregivers could be asked to do the same to screen for distress. At present, referrals for therapy as needed are a standard part of care for HNC survivors [38]. It stands to reason that routine assessment of mental health and subsequent referrals for caregivers are warranted as well.

We also consider the possibility that differences in perception of patient’s QOL signify an unmet need for communication between patients and their caregivers. In a series of semi-structured interviews, Badr et al. reported that communication was often diminished because patients felt poor or wanted to shield their partners from cancer-related concerns [10]. Outside of HNC, such failures at communication have been shown to have deleterious consequences for caregiver well-being. In a study of 193 severely ill patients (i.e., suffering from cancer, COPD, or congestive heart failure) and their caregivers, 39.9% of caregivers desired more communication with their patient regarding their needs and goals for treatment, and those who complained of difficulties communicating had higher caregiver burden (measured by Zarit Burden Inventory) [39]. This explanation would underscore the utility of interventions that target caregivers and their patients together. Specifically, medical providers could facilitate discussions in which patients and caregivers both express their thoughts regarding the treatment/recovery process and share how their own well-being is being affected. By making this a part of routine follow-up appointments, providers can take an active role in ensuring long-term communication between patients and caregivers, which may positively impact caregiver distress.

It is worth noting that not all of our caregivers disagreed with their patients regarding patient QOL. Indeed, a majority of dyads were concordant in their opinions which naturally begs the question, are there particular characteristics that make caregivers more likely to negatively perceive patients’ QOL? This particular study examined a number of clinical and demographic variables, though there are undeniably other sources of heterogeneity among patient-caregiver dyads, including family dynamics and specific caregiver responsibilities. Soliciting the specific opinions and experiences of disagreeing caregivers in semi-structured interviews like those conducted by Badr et al. [10] may be an effective way of delineating predisposing characteristics.

Limitations of this study warrant discussion. Most notably, the sample size for this study limits its ability to determine significance, and future studies should reexamine our findings with larger cohorts. Moreover, interrelationships with variables that were not examined in this study possibly exist.

## Conclusions

At present, caregiver burden in the context of HNC remains relatively understudied. To our knowledge, this is the first study to reveal that HNC caregivers who perceive patients’ QOL more negatively than patients themselves in the post-treatment period have significantly greater caregiver distress. Specifically, caregivers who believe their patients’ QOL is worse than patients believe themselves score significantly higher on the PHQ8 than those who do not disagree with their patients. We propose caregiver-targeted interventions, including screening for differences in perception of patient QOL to identify distressed caregivers and referrals for therapy as needed. Additionally, we propose interventions that target both patients and caregivers together, including provider facilitated discussions between patients and caregivers at routine appointments to ensure adequate communication. These strategies should be examined in future studies. As efforts lead to effective interventions, improvement in long-term outcomes for patients and their caregivers is likely to be seen.

## Supplementary Information

**Additional file 1.**

## Data Availability

The data that support the findings of this study are available on reasonable request from the corresponding author M.L.N. The data are not publicly available due to them containing information that could compromise research participant privacy/consent.

## Abbreviations

HNC: Head and Neck Cancer
QOL: Quality of Life
UWQOL: University of Washington Quality of Life Questionnaire
PHQ-8: Patient Health Questionaire-8
GAD-7: Generalized Anxiety Disorder Questionaire-7

## References

[1] Ezra EC, Samuel JL, Nicole LE (2016). American cancer society head and neck cancer survivorship care guideline. CA Cancer J Clin.

[2] Longacre ML, Ridge JA, Burtness BA, Galloway TJ, Fang CY (2012). Psychological functioning of caregivers for head and neck cancer patients. Oral Oncol.

[3] Vickery LE, Latchford G, Hewison J, Bellew M, Feber T (2003). The impact of head and neck cancer and facial disfigurement on the quality of life of patients and their partners. Head Neck.

[4] King SN, Dunlap NE, Tennant PA, Pitts T (2016). Pathophysiology of radiation-induced dysphagia in head and neck cancer. Dysphagia.

[5] Porter LS, Keefe FJ, Garst J (2010). Caregiver-assisted coping skills training for lung cancer: results of a randomized clinical trial. J Pain Symptom Manag.

[6] Pruchno RA, JMichaels JE, Potashnik SL (1990). Predictors of institutionalization among Alzheimer disease victims with caregiving spouses. J Gerontol.

[7] Posluszny DM, Dougall AL, Johnson JT (2015). Posttraumatic stress disorder symptoms in newly diagnosed patients with head and neck cancer and their partners. Head Neck.

[8] Sterba KR, Zapka J, Armeson KE, Shirai K, Buchanan A, Day TA, Alberg AJ (2017). Physical and emotional well-being and support in newly diagnosed head and neck cancer patient-caregiver dyads. J Psychosoc Oncol.

[9] Hanly P, Maguire R, Balfe M (2016). Burden and happiness in head and neck cancer carers: the role of supportive care needs. Support Care Cancer.

[10] Badr H, Herbert K, Reckson B, Rainey H, Sallam A, Gupta V (2016). Unmet needs and relationship challenges of head and neck cancer patients and their spouses. J Psychosoc Oncol.

[11] Balfe M, Maguire R, Hanly P (2016). Distress in long-term head and neck cancer carers: a qualitative study of carers’ perspectives. J Clin Nurs.

[12] Lee Y, Lin PY, Chien CY, Fang FM (2015). Prevalence and risk factors of depressive disorder in caregivers of patients with head and neck cancer. Psychooncology.

[13] Vanderwerker LC, Laff RE, Kadan-Lottick NS (2005). Psychiatric disorder and mental health service use among caregivers of advanced cancer patients. J Clin Oncol.

[14] Lang PO, Zekry D, Michel JP (2010). Early markers of prolonged hospital stay in demented inpatients: a multicenter and prospective study. J Nutr Health Aging.

[15] Mittelman MS, Ferris SH, Shulman E, Steinberg G, Levin B (1996). A family intervention to delay nursing home placement of patients with Alzheimer disease. A randomized controlled trial. JAMA.

[16] Yaffe K, Fox P, Newcomer R (2002). Patient and caregiver characteristics and nursing home placement in patients with dementia. JAMA.

[17] Lluis Conde-Sala J, Garre-Olma J, Turro-Garriga O (2009). Factors related to perceived quality of life in patients with Alzheimer’s disease: the patient’s perception compared with that of caregivers. Int J Geriatr Psychiatry.

[18] Moon H, Townsend AL, Whitlatch CJ, Dilworth-Anderson P (2017). Quality of life for dementia caregiving dyads: effects of incongruent perceptions of Everday care and values. The Gerentologist.

[19] Twiddy M, House A, Jones F (2012). The association between discrepancy in illness representations on distress in stroke patients and carers. J Psychosom Res.

[20] McCarthy MJ, Lyons KS (2015). Incongruence between stroke survivor and spouse perceptions of survivor functioning and effects on spouse mental health: a mixed-methods pilot study. Aging Ment Health.

[21] Miaskowski C, Zimmer EF, Barrett KM, Dibble SL, Wallhagen M (1997). Differences in patients’ and family caregivers’ perception of the pain experience influence patient and caregiver outcomes. Pain.

[22] Cano A, Corley AM, Clark SM, Martinez SC (2017). A couple-based psychological treatment for chronic pain and relationship distress. Cogn Behav Pract.

[23] Lyons KS, Miller LM, McCarthy MJ (2016). The roles of dyadic appraisal and dyadic coping in couples with lung cancer. J Fam Nurs.

[24] Merz EL, Malcarne VL, Ko CM (2011). Dyadic concordance among prostate cancer patients and their partners and health-related quality of life: does it matter?. Psychol Health.

[25] Kroenke K, Strine TW, Spitzer RL, Williams JBW, Berry JT, Mokdad AH (2009). The PHQ-8 as a measure of current depression in the general population. J Affect Disord.

[26] Lowe B, Decker O, Müller S (2008). Validation and standardization of the generalized anxiety disorder screener (GAD-7) in the general population. Med Care.

[27] Plummer F, Manea L, Trepel D, McMillan D (2016). Screening for anxiety disorders with the GAD-7 and GAD-2: a systematic review and diagnostic metanalysis. Gen Hosp Psychiatry.

[28] Reyes-Gibby CC, Anderson KO, Morrow PK, Shete S, Hassan S (2012). Depressive symptoms and health-related quality of life in breast cancer survivors. J Womens Health.

[29] Rogers SN, Lowe D, Yueh B, Weymuller EA (2010). The physical function and social-emotional function subscales of the University of Washington quality of life questionnaire. Arch Otolaryngol Head Neck Surg.

[30] El-Deiry MW, Futran ND, McDowell JA, Weymuller EA, Yueh B (2009). Influences and predictors of long-term quality of life in head and neck cancer survivors. Arch Otolaryngol Head Neck Surg.

[31] Vartanian J, Carvalho A, Yueh B (2004). Long-term quality-of-life evaluation after head and neck cancer treatment in a developing country. Arch Otolaryngol Head Neck Surg.

[32] Nilsen ML, Mady LJ, Hodges J, Wasserman-Wincko T, Johnson JT (2019). Burden of treatment: reported outcomes in a head and neck cancer survivorship clinic. Laryngoscope.

[33] Lee C, Lee Y, Wang L, Chien C, Fang F, Lin P (2017). Depression, anxiety, quality of life, and predictors of depressive disorders in caregivers of patients with head and neck cancer: a six-month follow-up study. J Psychosom Res.

[34] Deschler DG, Walsh K, Hayden RE (2004). Follow-up quality of life assessment in patients after head and neck surgery as evaluated by lay caregivers. ORL Head Heck Nurs.

[35] Hodges LJ, Humphris GM (2009). Fear of recurrence and psychological distress in head and neck cancer patients and their carers. Psychooncology.

[36] Richardson AE, Morton RP, Broadbent EA (2015). Caregivers’ illness perceptions contribute to quality of life in head and neck cancer patients at diagnosis. J Psychosoc Oncol.

[37] Richardson AE, Morton RP, Broadbent EA (2016). Changes over time in head and neck cancer patients’ and caregivers’ illness perceptions and relationships with quality of life. Psychol Health.

[38] Nguyen NA, Ringash J (2018). Head and neck survivorship care: a review of the current guideline and remaining unmet needs. Curr Treat Options Oncol.

[39] Fried TR, Bradley EH, O'Leary JR, Byers AL (2005). Unmet desire for caregiver-patient communication and increased caregiver burden. J Am Geriatr Soc.

