# Supplementary material for: Disparity of perception of quality of life between head and neck cancer patients and caregivers
Source: BMC Cancer. 2021 Oct 20;21:1127. doi: 10.1186/s12885-021-08865-7 (PMC8527693; doi:10.1186/s12885-021-08865-7)

Supplementary Figure 1. Simple slope analysis for interaction between Difference in Social-Emotional Impairment and Difference in Physical Impairment for the Generalized Anxiety Disorder Questionaire-7 (GAD-7) and Patient Health Questionaire-8 (PHQ-8)


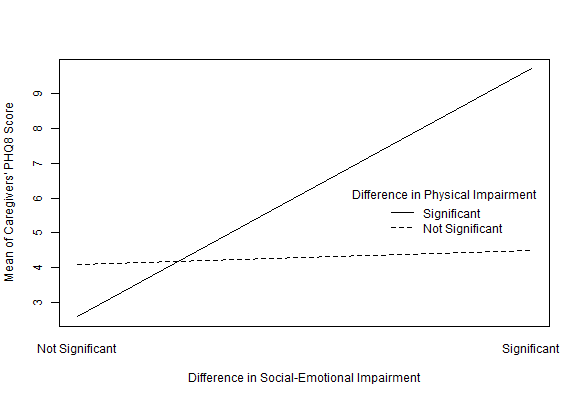

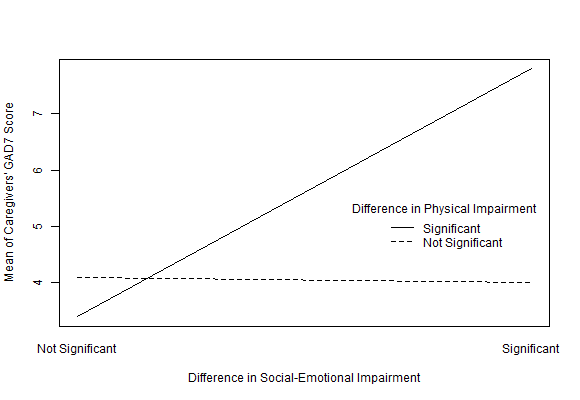

Supplement: Supplementary file 1 — Additional file 1. [file 12885_2021_8865_MOESM1_ESM.docx]
